# A Haloarchaeal Transcriptional Regulator That Represses the Expression of CRISPR-Associated Genes

**DOI:** 10.3390/microorganisms12091772

**Published:** 2024-08-27

**Authors:** Israela Turgeman-Grott, Yarden Shalev, Netta Shemesh, Rachel Levy, Inbar Eini, Metsada Pasmanik-Chor, Uri Gophna

**Affiliations:** 1 Shmunis School of Biomedicine and Cancer Research, George S. Wise Faculty of Life Sciences, Tel Aviv University, Tel Aviv 6997801, Israel; 2 Bioinformatics Unit, George S. Wise Faculty of Life Sciences, Tel Aviv University, Tel Aviv 6997801, Israel

**Keywords:** haloarchaea, archaea, regulation, transcription, CRISPR-Cas, csa3a, csa, crr, pHM500, Type I-B

## Abstract

Clustered regularly interspaced short palindromic repeats (CRISPR)-Cas (CRISPR-associated proteins) systems provide acquired heritable protection to bacteria and archaea against selfish DNA elements, such as viruses. These systems must be tightly regulated because they can capture DNA fragments from foreign selfish elements, and also occasionally from self-chromosomes, resulting in autoimmunity. Most known species from the halophilic archaeal genus *Haloferax* contain type I-B CRISPR-Cas systems, and the strongest hotspot for self-spacer acquisition by *H. mediterranei* was a locus that contained a putative transposable element, as well as the gene *HFX_2341*, which was a very frequent target for self-targeting spacers. To test whether this gene is CRISPR-associated, we investigated it using bioinformatics, deletion, over-expression, and comparative transcriptomics. We show that *HFX_2341* is a global transcriptional regulator that can repress diverse genes, since its deletion results in significantly higher expression of multiple genes, especially those involved in nutrient transport. When over-expressed, *HFX_2341* strongly repressed the transcript production of all *cas* genes tested, both those involved in spacer acquisition (*cas1*, *2* and *4*) and those required for destroying selfish genetic elements (*cas3* and *5–8*). Considering that *HFX_2341* is highly conserved in haloarchaea, with homologs that are present in species that do not encode the CRISPR-Cas system, we conclude that it is a global regulator that is also involved in *cas* gene regulation, either directly or indirectly.

## 1. Introduction

Clustered regularly interspaced short palindromic repeats (CRISPR)-Cas (CRISPR-associated) systems primarily provide acquired heritable protection to bacteria and archaea against invasion by selfish DNA elements, such as viruses [1,2]. These systems can capture DNA fragments from foreign selfish elements and integrate these fragments into their CRISPR arrays [3,4]. The DNA in the CRISPR array is then transcribed and processed into small crRNA [5]. In class I type I systems, these small crRNAs are then loaded onto a protein complex, called CASCADE, and when this protein-RNA complex recognizes a DNA sequence that matches the crRNA, the nuclease-helicase Cas3 is recruited and starts degrading the foreign DNA, a process known as “interference” [5]. Since cells must always be ready to face viruses and other molecular invaders, the “interference” machinery is expressed at a substantial basal level, providing some level of defense against DNA-based parasites. Nonetheless, genes involved in interference may be induced to even higher levels upon certain triggers, such as infection by a virus.

In contrast, the genes involved in the acquisition of new spacers (sometimes known as “adaptation”) are often expressed at very low levels [5]. Most known species from the halophilic archaeal genus *Haloferax* contain type I-B CRISPR-Cas systems, and two of them, *H. volcanii* and *H. mediterranei,* were shown to be capable of new spacer acquisition from self-chromosomes, especially following mating by cell fusion [6,7]. It is therefore not surprising that spacer acquisition is tightly regulated in those species and induced only by specific conditions, such as interspecies mating [6]. The strongest hotspot for self-spacer acquisition by *H. mediterranei* was a locus surrounding genome coordinates 2306000–2307000 that contained a putative transposable element, as well as the gene *HFX_2341*, which was a very frequent target for self-targeting spacers. This gene is annotated in NCBI as a DUF6293 (domain of unknown function 6293) family protein. To test whether this gene is CRISPR-associated, we investigated it using bioinformatics, deletion, over-expression, and comparative transcriptomics.

## 2. Results

HFX_2341 is currently annotated as a 308 amino acid-long hypothetical protein encoded on the *H. mediterranei* main chromosome. Notably, the *cas* genes of both *H. volcanii* [8] and *H. mediterranei* are plasmid-encoded [9], and the *HFX_2341* gene is ~178 kb from the nearest CRISPR array in the genome, and thus does not have a genomic association with CRISPR. A BLASTP sequence similarity search yielded many homologs from other archaeal species, especially other haloarchaea, including a full-length homolog with >72% identity in the CRISPR-negative species *Halobacterium salinarum* NRC-1, raising doubts about its potential association with CRISPR. 

To gain insights into HFX_2341 function using bioinformatics, we used HHpred analysis [10] that is based on secondary and tertiary structural similarity between proteins that may not be similar in sequence. This analysis revealed that HFX_2341 has a significant structural similarity to several CRISPR-associated proteins from the archaeal phylum Thermoproteota. The strongest structural similarity (probability of 99.74% and E-value of 6.6 × 10^−16^) was to Csa3a, a CRISPR-associated transcriptional regulator, from the thermoacidophilic archaeon *Saccharolobus* (formerly known as *Sulfolobus*) *solfataricus* (Sso1445, PDB structure 2WTE) [11]. This protein is thought to be activated by binding to cyclic oligoadenylates [12]. A strong similarity was also observed to Sso1393 from that species, which is a CRISPR-associated ring nuclease that has been shown to degrade cyclic oligoadenylates [13]. The strong structural conservation of the C-terminal DNA-binding domain to multiple transcriptional repressors indicates that the protein is highly likely to regulate transcription, perhaps in response to a cyclic adenylate signal.

**Figure 1:**
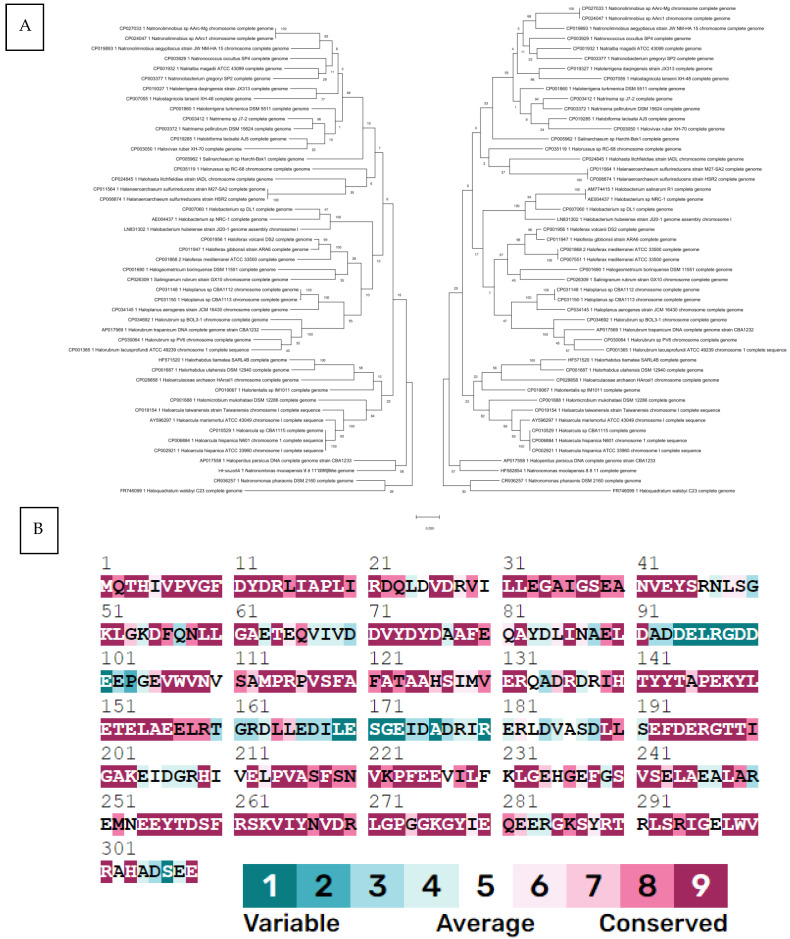
(**A**) Molecular phylogenetic analysis of the *HFX_2341* gene (left tree) and *rpoB1* gene (right tree) using maximum likelihood. The evolutionary history was inferred by using the maximum likelihood method based on the Tamura–Nei model [23]. The tree with the highest log likelihood (−18,940.96 for *HFX_2341* and −18,940.87 for *rpoB1*) is shown. The percentage of trees in which the associated taxa clustered together is shown next to the branches. Initial tree(s) for the heuristic search were obtained automatically by applying Neighbor-Join and BioNJ algorithms to a matrix of pairwise distances estimated using the maximum composite likelihood (MCL) approach, and then selecting the topology with superior log likelihood value. The tree is drawn to scale, with branch lengths measured in the number of substitutions per site. This analysis involved 47 nucleotide sequences for *HFX_2341* and 49 for *rpoB1*. There were a total of 1008 positions for each of the final dataset. Evolutionary analyses were conducted using MEGA X [24]. (**B**) ConSurf [15] conservation profile for HFX_2341 protein. The residues in the sequence are colored by their conservation grades using the nine-grade color-coding bar [25], indicating that most residues are highly conserved.

To determine the evolutionary history of *HFX_2341,* we constructed a phylogenetic tree and compared it to a tree of a well-established phylogenetic marker gene for Halobacteriales, *rpoB1*, a conserved subunit of RNA polymerase [14]. This comparison indicated that *HFX_2341* shows a strong vertical signal, similar to that of RpoB1, and has not been horizontally transferred often (Figure 1A). ConSurf analysis [15] showed that many Csa3a residues are highly conserved in archaeal orthologs (Figure 1B).

Since *H. mediterranei* has extremely low mRNA levels of the *CRISPR-cas* genes (average of 22.67 RPKM, [16]) and Csa3a homologs that are structurally similar to HFX_2341 were associated with regulation of *cas* genes in *S. solfataricus* and *Sulfolobus islandicus* [17,18,19,20,21], we tested whether HFX_2341 in *H. mediterranei* has an effect on the expression of *cas* genes. To that end, we generated a Δ*HFX_2341* knockout strain and compared its global mRNA levels to those of the wild-type using RNA-Seq (Materials and Methods). RNA-Seq results showed no statistically significant differences in the *cas* gene expression between Δ*HFX_2341* and the wild type.

**Figure 2:**
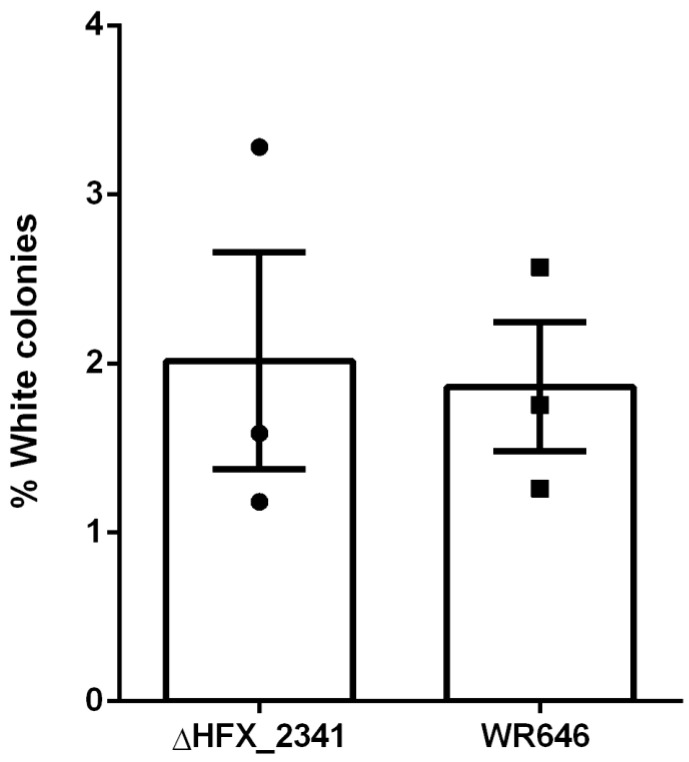
Fraction of white colonies obtained after self-targeting of the *crtI* chromosomal gene using the CRISPR-interference machinery in *H. mediterranei* control (WR646) and ΔHFX_2341 strains (N = 3). Each column represents the mean with the denotation of the SEM bars.

To further investigate if CRISPR interference would be enhanced in the *H. mediterranei* ΔHFX_2341 strain, we performed a self-targeting assay as previously described in *H. volcanii* [22] that tests the activity of the CRISPR-Cas interference machinery. In this assay (see also Materials and Methods), the fraction of colonies that lose their pink pigmentation and become white serves as a proxy for CRISPR-Cas activity when expressing a specific crRNA against a self-gene (*crtI*), which is required for the pink (wild-type) phenotype. The results of this self-targeting assay showed only a minor increase in self-targeting of the ΔHFX_2341 strain compared to the wild-type control (Figure 2), and indicated that *HFX_2341* mRNA did not result in an increase in CRISPR interference levels.

**Figure 3:**
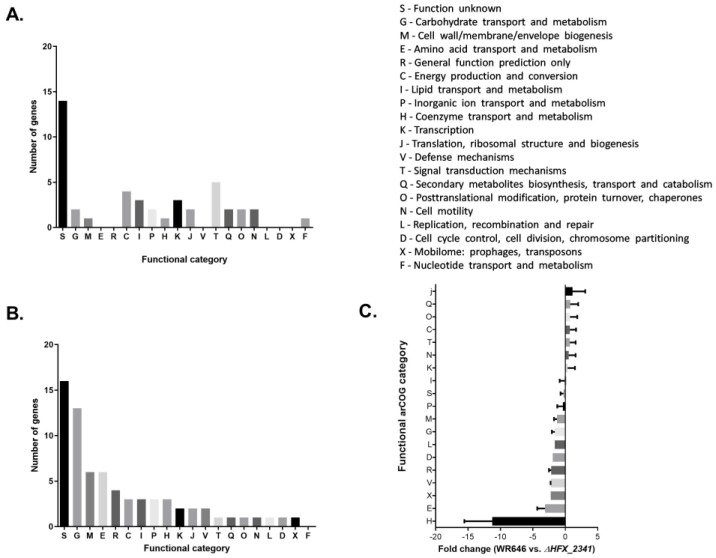
RNA-seq results of the *H. mediterranei* wild-type vs. the ΔHFX_2341 strain. Significantly changed mRNA levels genes using ANOVA < 0.05, FC > 1.5 (Supplementary Table S2) are presented. The results presented are the mean of three biological replicates for the wild-type strain and four biological replicates for the ΔHFX_2341 strain. (**A**) Up-regulated genes organized by functional categories (wild-type/ΔHFX_2341). (**B**) Down-regulated genes organized by functional categories (wild-type/ΔHFX_2341). (**C**) Fold change average according to the functional categories of the genes. Means with SEM are presented.

In contrast to the modest changes in the mRNA levels of *cas* genes, multiple housekeeping genes did differ in gene expression in the ΔHFX_2341 mutant. Some genes appeared to be up-regulated by HFX_2341, most of them associated with signal transduction mechanisms (category T) and energy production or conversion (category C), but the majority of the significantly changed genes were down-regulated by HFX_2341, belonging to functional categories such as carbohydrate transport and metabolism (category G), cell wall/membrane/envelope biogenesis (category M), and amino acid transport and metabolism (category E). Most of the genes that had an altered level of expression were of unknown function (Figure 3A,B, Supplementary Tables S1 and S2). The biggest difference was observed in the functional categories of transport and metabolism (mean of 3-fold lower in the wild-type) of amino acids and coenzymes, which were up-regulated in the mutant (mean of 11-fold lower in the wild-type) (Figure 3C).

**Figure 4:**
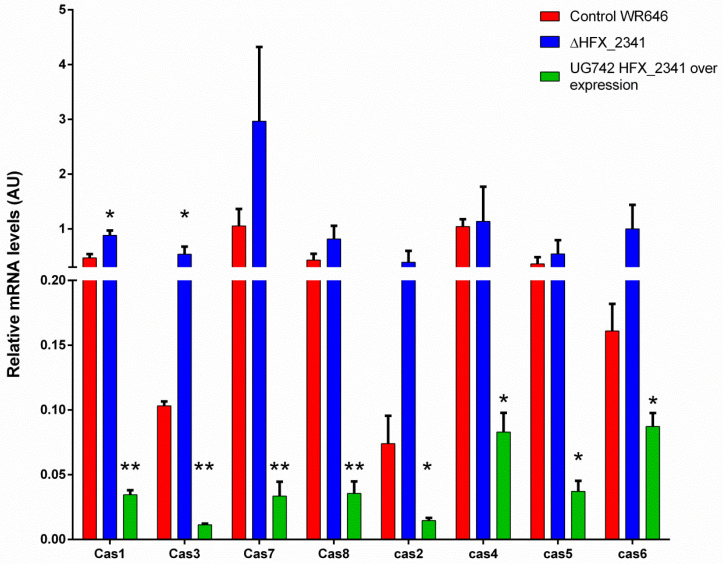
Quantification of the *CRISPR-cas* mRNA levels from *H. mediterranei* in the control (WR646), Δ*HFX_2341* strain, and HFX_2341 over-expression (N > 4). All levels are normalized to the expression of a housekeeping gene (*polB*) in the respective strains. Each column represents the mean with the denotation of the SEM bars. * = *p*-value < 0.05; ** = *p*-value < 0.01, two-sided Mann–Whitney test compared to wild type. AU—arbitrary units.

It is known that large-scale analysis is often not sensitive enough to detect small changes in mRNA expression. In order to further investigate if HFX_2341 has an effect on *cas* gene expression, we measured the mRNA levels of the *cas* genes using quantitative PCR (QPCR). The QPCR results showed increased levels of mRNA for all *cas* genes in the ΔHFX_2341 strain in comparison to the control (Figure 4). Statistically significant changes were observed for *cas1,* a gene involved in the acquisition of new spacers (about 1.8-fold increase in average mRNA levels), and especially for *cas3*, a gene involved in CRISPR interference (5.2-fold average increase), which generally has basal levels much higher than *cas1*. When we over-expressed *HFX_2341,* this strongly and significantly reduced the mRNA expression of these *cas* genes, as quantified by QPCR (Figure 4), supporting its role as transcriptional repressor of these genes. Taken together, these results indicate that in wild-type cells, basal levels of *HFX_2341* correspond to basal levels of *cas* gene expression and thus its de-repression yields only modest up-regulation of expression, while raising the levels of this repressor results in a much stronger down-regulation effect. 

## 3. Discussion

While HFX_2341 has homologs in most haloarchaeal genomes (643/861, genomes in NCBI July 2024, when requiring 75% coverage), its function has not been investigated to date. We identified its structural similarity to CRISPR-associated proteins in members of the order Sufolobales and suspected it to be responsible for the low mRNA levels of the *cas* genes in *H. mediterranei,* since its C-terminal domain resembles multiple transcriptional repressors. However, deletion analysis showed that the mRNA levels of the *cas* genes are very low in the Δ*HFX_2341* strain as well as in the wild type. Over-expressing the Δ*HFX_2341* gene on a plasmid led to a substantial further reduction of the *cas* gene mRNA levels. From these results, it is clear that HFX_2341 is not the only regulator responsible for the low *cas* gene expression in *H. mediterranei*, even though it clearly has a negative effect on their mRNA levels when over-expressed. We show that HFX_2341 acts as a general transcriptional regulator that strongly affects many metabolic genes and genes of unknown function. This could explain its presence in CRISPR-negative strains, such as *H. salinarum* NRC-1. HFX_2341 probably acts as a repressor, since in most cases its presence leads to lower gene expression in the wild type compared to the Δ*HFX_2341* mutant. Most of the genes affected by the deletion are involved in transport of carbohydrates, lipids, ions, co-enzymes, and amino acids, but some are also associated with biogenesis of the cell envelope, cell division, and defense mechanisms. This is in contrast to its structural analog in *S. islandicus,* which is a more specific transcriptional activator of DNA repair genes and the CRISPR acquisition machinery [20,21].

An obvious question that immediately comes to mind is whether HFX_2341 is a transcriptional repressor that is activated in response to a cytoplasmic signal molecule (secondary messenger), as implied by the strong structural similarity to the *Sulfolobus* Csa3a. If so, what is the molecule that it senses? While there is no homolog of the adenylate cyclase family associated with-type III CRISPR-Cas systems [26,27] in *H. mediterranei*, this does not preclude the existence of a different enzyme capable of converting ATP into cyclic tetra- or hexa-adenylate. An alternative is that the signal molecule in question may be cyclic AMP or cyclic di-AMP, which have recently been shown to be produced by, and essential in, *H. volcanii* [28]. Furthermore, over-expression of DacZ in *H. volcanii* was toxic to the cells, indicating that levels of, importantly, the diadenylate cyclase DacZ are not just highly conserved in haloarchaea, but also coupled with the sensing of osomotic pressure [28], and thus the level of the signal produced likely responds to environmental stimuli, which is consistent with repression of multiple metabolic genes and transporters by HFX_2341 as a potential response regulator. However, this prediction clearly requires much experimental validation, which should be undertaken in *H. volcanii*, in which genetics is simpler and an inducible *dacZ* gene already exists. 

An important limitation of our study is that both the role of HFX_2341 as a global regulator and its more specific effect on the expression of *cas* genes could be indirect and mediated by downstream regulatory factors. Our analysis should thus be followed up by identification of the regulatory regions to which HFX_2341 binds, through approaches such as CHIP-Seq. Given its DNA-binding domain and the effects its deletion has on the mRNA levels of multiple genes, we suggest renaming *HFX_2341* to *crr* (**CRISPR-related repressor**).

## 4. Materials and Methods

### 4.1. Strains and Plasmids 

The strains and plasmids used in this study are described in Table 1.

### 4.2. Culture Conditions

*H. mediterranei* cells were routinely grown in the rich medium Hv-YPC (*Haloferax volcanii*–yeast extract, peptone, casamino), containing 30% SW (salt water: 144 g of NaCl, 21 g of MgSO_4_ · 7H_2_O, 18 g of MgCl_2_ · 6H_2_O, 4.2 g of KCl, and 12 mM Tris HCl (pH 7.5), per liter). For solid media, agar (Difco, Sparks, MD, USA) was added at a concentration of 15 g per liter. The media was heated on a hot plate until dissolved. Yeast extract (0.5%, wt/vol; Difco), 0.1% (wt/vol) peptone (Oxoid, Basingstoke, UK), and 0.1% (wt/vol) casamino acids (Difco) were added, and the medium was autoclaved. After cooling, CaCl_2_ was added to a final concentration of 3 mM. Strain UG742 was routinely maintained in casamino acid medium (Hv-Ca), which was made in a manner similar to Hv-YPC, except that peptone and yeast extract were not added and casamino acids powder was added to a final concentration of 0.5% (wt/vol). For plasmid maintenance, casamino acids medium (without yeast extract) was used.

The Hv-Min minimal medium (0.1 mM) contained the same concentration of salts as Hv-YPC, except that Tris HCl (pH 7.5) was added to a concentration of 42 mM. After autoclaving and cooling, 4.25 mL of a sodium dl-lactate solution (60%, wt/vol), 3.83 g of disodium succinic acid · 6H_2_O, 0.25 mL of glycerol, 5 mL of a 1 M NH_4_Cl solution, 6 mL of a 0.5 M CaCl_2_ solution, 1 mL of a trace element solution, 0.8 mg of thiamine, and 0.1 mg of biotin were added per liter of Hv-Min. Potassium phosphate buffer (pH 7.5) was supplemented to a final concentration of 0.1 mM phosphate (the only phosphate source). 

The p.*tnaA* promoter of UG742 was activated by transferring the liquid culture of that strain to Hv-YPC supplemented with tryptophan to a final concentration of 2 mM about 12 h before RNA was extracted for quantitative PCR. 

As per the experimental requirements, we added either thymidine or hypoxanthine to a final concentration of 40 μg/mL, along with one of the following at 50 μg/mL: leucine, tryptophan, uracil, methionine, glycine, or pantothenic acid to the growth media. 

Then, 5-fluoroorotic acid (5-FOA) and uracil were used to prepare the pop-out selection medium, with final concentrations of 50 μg/mL and 10 μg/mL, respectively.

### 4.3. Transformation of Haloferax Mediterranei 

Transformation of *H. mediterranei* was carried out using the PEG method as described in [22]. 

Culture Preparation and Cell Harvesting: Cells were grown in 10 mL culture to OD650 ≈ 0.8. The culture was pelleted by centrifugation at 4500× *g* (6000 rpm) for 8 min at 25 °C. All subsequent steps were performed at room temperature.

Cell Resuspension and Spheroplast Formation: The pellet was gently resuspended in 2 mL spheroplasting solution (1 M NaCl, 27 mM KCl, 50 mM Tris.HCl pH 8.5). The suspended cells were re-pelleted at 4500× *g* for 8 min. The pellet was carefully resuspended in 200 µL of spheroplasting solution (200 µL per transformation), followed by adding 20 µL of 0.5M EDTA (pH 8.0) to the tube and mixing slowly by inversion. The mixture was incubated at room temperature for 10 min to form spheroplast cells.

DNA Preparation and Transformation: During spheroplast formation, DNA samples were prepared in a total volume of 30 µL: 10 µL of DNA (~1–2 µg or 10 µL of dH_2_O (as a control), 15 µL of spheroplasting solution, and 5 µL of 0.5 M EDTA (pH 8.0)). After the 10-min spheroplast formation, the prepared DNA was added to the spheroplasts, as done with EDTA. The mixture was incubated at room temperature for 5 min.

PEG-Mediated Transformation: 250 µL of 60% PEG600 (160 µL of PEG 600 (SIGMA, Livonia, MI, USA) with 200 µL of spheroplasting solution) was added to the transformation reaction and incubated at room temperature for at least 30 min.

Post-Transformation Processing: 1.5 mL of regeneration solution (23% SW, 15% sucrose, 3.75 mM CaCl_2_) was added, mixed by inversion, and incubated at room temperature for 2 min. Cells were pelleted by centrifugation at 4500× *g* for 8 min at 25 °C and the supernatant was removed. 

Cell Regeneration and plating: 1 mL of YPC-sucrose solution (18% SW, 0.5% yeast extract, 0.1% peptone, 0.1% casamino acids, 15% sucrose, 3 mM Cacl2) was added, and the whole pellet was transferred to a 4 mL sterile tube, avoiding resuspension. The tube was incubated undisturbed at 45 °C for 1.5–2 h. The pellet was then resuspended by tapping the side of the tube and returned to 45 °C for an additional 3–4 h with rotation. For final processing and plating, the cells were transferred to a 2 mL round-bottomed tube and pelleted at 4500× *g* for 8 min at 25 °C. The supernatant was removed, and the pellet was gently resuspended in 1 mL Hv-YPC. The cells were then diluted and plated on appropriate Hv-YPC/ Hv-Ca/ or Hv-Min selectable media plates. 

### 4.4. Gene Knockout of Haloferax Mediterranei

Gene knockout of *H. mediterranei* was carried out using the pop-in/pop-out method as described in [32], using ‘suicide vector’ pTA131 [29], having the *pyrE*2 selectable genetic marker with ~1000 bp flanking regions to a selected region to be deleted, in this case in the *H. mediterranei HFX_2341* gene (see Supplementary File S1). The suicide vector was cloned using *Hin*dIII, *Bam*HI, and *Kpn*I restriction enzymes (FastDigest by Thremo Fisher, Waltham, MA, USA). The two segments of the DNA insert were cut and ligated (T4 DNA ligase by Themo Fisher), and the product was amplified by PCR (Phusion High-Fidelity DNA polymerase by Themo Fisher). The primers used for the plasmid construction were (restriction sites are capitalized) forward up (HindIII) aaaaAAGCTTtgaaacgtgggaccgaacaa, reverse up (KpnI) aaaaGGTACCggcgagttgtgggtccgc, forward down (KpnI) aaaaGGTACCctcgacgttcgcttcgctcccgatag, and reverse down (BamHI) aaaaGGATCCcgggacatggatgattgcca. 

### 4.5. Phylogenetic and Conservation Analysis of HFX_2341 

The protein and nucleotide sequences of HFX_2341 and RpoB1 from *H. mediterranei* were retrieved from the Kyoto Encyclopedia of Genes and Genomes (KEGG) database [33]. The nucleotide sequences of these genes from different haloarchaea that were shown to have *HFX_2341* gene homologs by BLASTN were retrieved from the National Center for Biotechnology Information (NCBI) database. Gene sequences were aligned using MAFFT [34]. Trees were constructed using maximum likelihood as implemented in MEGA [24], with 100 bootstrap trials. The evolutionary history was inferred from the nucleotide sequences using the maximum likelihood method, based on the Tamura–Nei model [23]. The tree with the highest log likelihood is shown in all phylogeny figures. Initial tree(s) for heuristic searches were obtained automatically by applying Neighbor-Join and BioNJ algorithms to a matrix of pairwise distances, estimated using the maximum composite likelihood (MCL) approach, and then by selecting the topology with the superior log likelihood value. The evolutionarily conserved amino acids of the *HFX_2341* protein were predicted by the ConSurf server [15].

### 4.6. Self-Targeting Assay

ΔHFX_2341 and WR646 were successfully transformed with UG479 (a self-targeting CRISPR plasmid). Three colonies, from each strain, with the plasmid were grown for a week in low Phosphate (0.1 mM) Hv-Min medium broth (42 °C, shaking conditions), before plated on YPC plates with two technical replicates. After growth and pigmentation accumulation, pink/white colony ratio was established. 

### 4.7. RNA Extraction

Total RNA was extracted from five biological replicates of the two strains (WR646 and Δ*HFX_2341*). Liquid culture of each replicate was grown in Hv-YPC broth to an OD_600nm_ of 0.5–0.6 (logarithmic stage), and 2 mL of each sample was centrifuged at 300× *g* for five minutes. The pellet was resuspended in 1 mL of TRIzol reagent (Rhenium, Modi’in, Israel) before adding 200 µL of phenol-chloroform and mixing thoroughly. The mix was centrifuged at 12,000 rcf for 15 min, and tubes were placed on ice. The RNeasy kit (Qiagen, Hilden, Germany) protocol was preceded with 400 µL of the supernatant, according to manufacturer guidelines.

### 4.8. RNA Sequencing

Sequencing and library preparation was performed by the Core Genomics Facility team at the University of Illinois, Chicago. The Ovation Complete Prokaryotic RNAseq library system by NuGEN was constructed for a set of 10 total RNA samples (five biological replicates of the two strains, WR646 and Δ*HFX_2341*). Sequencing was done using Illumina HiSeq4000 2 × 150. Between 22 and 30 million reads were obtained per sample. 

### 4.9. RNA Sequencing Analysis

RNA-seq analysis was performed using Partek^®^ Flow^®^ (Build version 9.0.20.0804). Alignment was done using Bowtie [35] to Haloferax mediterranei ATCC 33500 reference genome; NCBI; https://ftp.ncbi.nlm.nih.gov/genomes/all/GCF/005/406/325/GCF_005406325.1_ASM540632v1/GCF_005406325.1_ASM540632v1_genomic.fna.gz, accessed on 15 May 2019). Aligned reads were filtered by minimum mapping quality 20. Quantification was performed on the annotation model using the Partek E/M algorithm [36] (https://ftp.ncbi.nlm.nih.gov/genomes/all/GCF/005/406/325/GCF_005406325.1_ASM540632v1/, accessed on 15 May 2019), followed by RPKM normalization [37]. A total of 3585 genes were detected and applied for GSA statistical analysis for differential expression (gene-specific analysis), followed by filtering (fold-change difference = 1.5 with pFDR < 0.05 (Supplementary Table S1) or ANOVA < 0.05 (Supplementary Table S2)). Analysis consists of 5 REP (ΔHFX_2341 biological replicates) and 4 GUG (WR646 biological replicates) samples (one WR646 sample was omitted from the analysis—UGU4—since it was a clear outlier). Function enrichment was determined using either the DAVID tool (Supplementary Table S1) [38] or arCOGs (Archaeal Clusters of Orthologous Genes) (Supplementary Table S2) [39].

### 4.10. Quantitative PCR

Cells were grown in YPC as described above, and RNA was extracted using four to seven cultures from each of the H. mediterranei strains: WR646, ΔHFX_2341, HFX_2341 over-expression after reaching OD600nm of 0.5–0.6. mRNA was reverse-transcribed using the iScript cDNA Synthesis Kit (Bio-Rad, Hercules, CA, USA) for cDNA synthesis. Quantitative PCR was performed on a C1000 Thermal Cycler (Bio-Rad) with qPCRBIO SyGreen Blue Mix Hi-ROX (PCRBIOSYSTEMS, London, UK). QPCR values obtained were normalized to the expression of a housekeeping gene (polB) in the respective strains.

## Figures and Tables

**Table 1:** Strains and plasmids used in this work.

Plasmids		
Name	Description	Source
pTA230		[29]
pTA231		[29]
pTA927		[30]
UG479	pMA-RQ-crRNA#3 [22] with crRNA#3 sequence replaced with (5′ to 3′) ggcgtagtcggcgtcgcttacgacctggtcacagag	This work
UG742	HFX_2341 cloned in pTA927 under the ptna promoter	This work
UG503	pTA131 with flanking regions spanning HFX_2341	This work
Strains		
Name		
WR646		[31]
Δ*HFX_2341*	WR646 Δ*HFX_2341*	This work
*HFX_2341 over-expression*	WR646 expressing *HFX_2341 from* UG742	This work

## Data Availability

The data supporting the conclusions of this article will be made available by the authors on request.

## Supplementary Materials

The following supporting information can be downloaded at: https://www.mdpi.com/article/10.3390/microorganisms12091772/s1, Figure S1: Structural similarity of HFX_2341 to Csa3a of *Sulfolobus solfataricus*; Table S1: Differentially expressed genes (fold-change difference>1.5) between the w.t. and Δ*HFX_2341* strain, with pFDR<0.05 gene, function according to the DAVID tool; Table S2: Differentially expressed genes between the w.t. and ΔHFX_2341 strain ANOVA<0.05 with functional arCOG category (Archaeal Clusters of Orthologous Genes).
