# Supplementary material for: A Haloarchaeal Transcriptional Regulator That Represses the Expression of CRISPR-Associated Genes
Source: Microorganisms. 2024 Aug 27;12(9):1772. doi: 10.3390/microorganisms12091772 (PMC11434293; doi:10.3390/microorganisms12091772)
Supplement: Supplementary file 1 [file microorganisms-12-01772-s001.zip › Supplementary Figure S1.pdf]

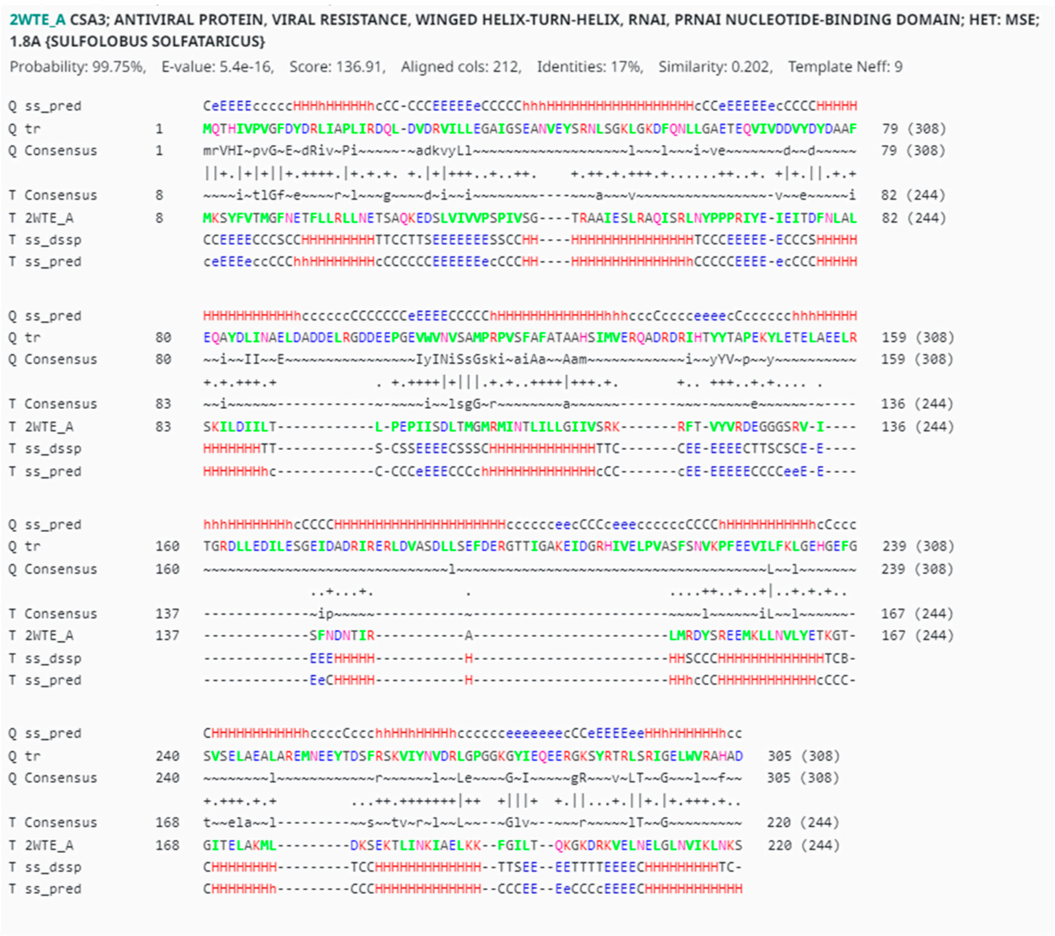

Supplementary Figure S1: Structural similarity of HFX_2341 to Csa3a of *Sulfolobus solfataricus*. HHpred, protein homology detection for HFX_2341 resulted in match with Sulfolobus Solfataricus csa3a protein, E. value= $5.4e^{-16}$.
